# Clinical Surgery of the Hospital of La Pitié

**Published:** 1843-01

**Authors:** 


### Art. III.

*Clinique Chirurgicale de l'Hôpital de la Pitié.* Par J. Lisfranc. Tome premier.—*Paris,* 1841. 8vo, pp. 696.

*Clinical Surgery of the Hospital of La Pitié.* By J. Lisfranc.—*Paris,* 1841.

We resume the analysis of this important work, left imperfect by the article in Number XXVII.

*Necrosis and Caries.* 1. Amputation is, as it ought to be, rare in the treatment of necrosis. Yet M. Lisfranc thinks that it is performed more frequently than necessity demands; and wisely inculcates a careful diagnosis as to the extent of the disease, before so harsh a remedy is actually resolved on. Though the discharge be copious, the fistulæ numerous, and the soft parts extensively involved, still the necrosis may be limited both in surface and in depth, and very far from requiring removal of the limb; in fact the source of all the secondary mischief may ultimately prove but little larger than the thumb-nail. Nature is adequate to the cure; we have only to assist her occasionally. When she has succeeded in fairly detaching the sequestrum, and is working towards its extrusion, we may be of signal service; previously and subsequently to this, we should be contented in most cases with practising "la médecine expectante." 2. Again we express delight and comfort at such sentiments as the following, emanating from Parisian surgery: "Be in no haste to operate, especially when there is no danger in delay. Let us limit anew the operative department of our profession; for never is surgery so beautiful and brilliant, as when obtaining a cure without the destruction of any organ, without plunging the bistoury into quivering flesh, and without causing the effusion of blood." Such words do credit alike to the heart and head. Would that the feeling was more strictly acted on! 3. But again have we to find fault with M. Lisfranc in regard to the absorption of sequestra. He now not only asserts plainly that such is possible, but maintains that it is of frequent occurrence; dragging in a certain M. Malespine as particeps criminis in his offence against sound surgery and common sense. A *dying* portion of bone may be partially reabsorbed, and may be wholly disintegrated by a vital process similar to what effects the major share of rapid ulceration in the soft tissues; but *dead* bone is liable neither to absorption nor disintegration, nor any other vital action; and moreover has but little prospect of being much altered by chemical or physical causes, until after its extrusion. Because M. Malespine finds an empty cavity in the interior of a bone, he has no right to announce forthwith, and on his own mere *ipsè dixit,* that this vacuity has been occasioned by death of the cancellated texture to a corresponding extent, and that the sequestrum has subsequently been wholly absorbed. Had there been a genuine sequestrum, that is, a dead and detached portion of bone, it would have remained cooped up in the interior, little altered if at all, until nature assisted by the surgeon had devised and accomplished means for its removal. Would it not be as easy, and infinitely more feasible, to suppose that the bone, which originally and normally occupied the place of this cavity, was not at once killed, and then eaten up by the absorbents at their leisure; but that by ulceration, preceded and assisted perhaps by interstitial absorption, it has been gradually disintegrated and removed, the disappearance of the osseous texture keeping pace with the advancement of the process? This process may advance more rapidly at certain periods and points than at others, and then small portions of bone may be actually necrosed, and lodge in the bottom of the cavity, unfavorably complicating the case. How often do we see caries and a shade of necrosis combined? But M. Malespine finding such portions, at once concludes that they are the debris or residuum of the large sequestrum, and triumphantly appeals to them as proof irrefragable of his theory: "The sequestrum was here and has been demolished; here are its bones!" But why should the absorption which devoured the large fragment so easily, tire of or choke on the fragments? A man who has eaten a whole cow need not worry on the tail, and certainly is expected not to permit fastidiousness of palate to spurn this ultimate morsel. But the truth is, the absorbents reject the large just as much as the small portion; they are not vultures, to feed on carrion; they are vivivorous cannibals, and live upon their next neighbours; they are given to habitual thirst, and will not hesitate to slake this on fluids animate or inanimate; but they will not touch solids which have ceased to possess the charm and the flavour of vitality. In short, by a vital process, just as different from necrosis as ulceration is from sloughing, the bone has been gradually reduced to a fluid condition; part of which fluid may have escaped externally, part may have been absorbed, and part remains in the cavity; this last containing not the debris of dead bone, but small individual sequestra, the formation of which has complicated the process, and which never will be absorbed, but are destined to be extruded along with the fluid in which they float, be that when it may. Such we hold to be the rational explanation of the internal cavities of M. Malespine. And once more we protest against such doctrine as he, aided and abetted by M. Lisfranc, has propounded. It is not only false in theory, but most dangerous in its practical tendency; inasmuch as he who leaves and wilfully deserts a loose sequestrum, already cooped up by substitute bone, in the hope that it shall be absorbed—and assists not nature to expel what is to all intents and purposes a foreign body—commits a grievous surgical error of omission, for which much active and skilful exertion subsequently may be unable fully to atone. 4. M. Malespine wishes to make out that tubercular degeneration of the cancellated texture of bone occurs much more rarely than is supposed. We hope he may be right. But still, supposing that he is so, it does not follow, as he infers, that the aforesaid cavities must be occasioned by necrosis. The interior of the bone may be and often is simply disintegrated, without any previous deposit of any tubercular or other morbid product. 5. In caries, cure is not to be hoped for until after removal of the carious portion by knife or cautery. But before proceeding to such an operation, it is not enough that we accurately diagnosticate the nature and extent of the degenerate ulcer. The surrounding soft parts are frequently the seat of an acute inflammatory action; and this must be subdued, before operative interference with the bone can be attempted with a just hope of success. 6. The actual cautery is preferred to the potential by M. Lisfranc. We disagree with him. The hot iron not only destroys the parts actually diseased, but likewise depresses the vitality of those immediately adjoining, thereby rendering them prone to the assumption of a carious action; and thus, after separation of the slough and sequestrum that follow the application, a carious instead of a healthy surface may be found beneath, if not immediately, at least long before the cicatrization of the wound. The potential cautery, of which the most convenient forms here are the chloride of zinc and the red oxide of mercury, destroys the original caries and does not predispose to the formation of a secondary. But when circumstances will permit, all caustics ought to be superseded by cutting instruments. 7. Messrs. Lisfranc and Malespine regard interstitial absorption of bone as of little moment, and as being merely the result of neighbouring suppuration of bone. On the contrary, observation has led us to believe that caries is *preceded* as well as attended by interstitial absorption, in and around the site of the principal disease; and that such a change of the bone is consequently always to be looked on with much suspicion, not on account of its own intrinsic merits, but as a precursor of one of the gravest forms of surgical malady. An open cancellated condition of the bone seems to be the essential nidus of caries. In the spongy heads, but little has to be done in paving the way for its accession; in the shafts and other sites of a laminated texture, however, the interstitial absorption has been in progress long before the actual caries appears. And in such situations we believe that caries has often been prevented by suitable treatment, (rest, counter-irritation, and attention to the system,) directed to the arrestment of this preliminary change in the normal texture of the bone. Again, then, we are not differing with our author on a mere point of theory, but on an important principle, highly available in practice.

*Nervous Ophthalmia.* The affection so named by M. Lisfranc is a good example of local *irritation* in contradistinction to an *inflammation;* in other words perverted action of the nerves, rather than of the blood-vessels of the part. The symptoms are lachrymation; no morbid appearance in the interior of the eye or on the cornea; marked photophobia; the eyelids carefully shut; on opening the eyelids, the patient feels as if points of nails were being driven into the globe; the conjunctiva is slightly reddened, but does not appear swollen. In the treatment, antiphlogistics fail. Extract of belladonna is to be rubbed on the temples and around the base of the orbits night and morning; sometimes the same medicine may be administered internally. In a few days the cure is complete. Finding such irritations of a mucous surface externally, closely simulating the state of inflammation, M. Lisfranc is naturally led to conclude that probably similar conditions and simulations may not unfrequently exist internally; and that many pains in the stomach, bowels, urethra, &c. supposed to be inflammatory, and yet resisting all antiphlogistic and revulsive means, may be dependent on mere irritation, and ready to yield almost at once to narcotics. We agree with M. Lisfranc in thinking this extremely probable.

A chapter on hard circumscribed tumours of the eyelids contains nothing new, and requires no comment.

*General rules regarding the extirpation and amputation of tumours.* By "extirpation" M. Lisfranc means removal of the tumour alone; by amputation, ablation of its investing integument also. We do not intend to follow him through all his "rules," many of them being trite enough, and chiefly connected with the mechanical department and its minutiæ; but shall briefly notice some axioms both interesting and important: 1. Having made a section of a tumour after its removal, it is very easy to determine its exact character, and to bring it under some item of a definite classification of such formations; but previously to operation how difficult is it for even the most experienced, in not a few cases, to predicate with accuracy the structure and tendency of the swelling. How often has hydrocele been confounded with sarcocele, and sarcocele returned the compliment? Chronic abscesses have been held to be solid formations, and their excision accordingly attempted; aneurisms have been thought abscesses, until the gush of arterial blood that followed the plunge of the bistoury, at once undeceived the surgeon, and filled him with consternation; Dupuytren mistook a fatty tumour in the orbit with a large vein on its front for an aneurism by anastomosis; Lisfranc cut down on what he supposed to be an exostosis, but which turned out to be a fibrous tumour of the soft parts; and we venture to say that the candid confession of every surgeon, however eminent—nay, the more eminent the more likely—would disclose many similar blunders in diagnosis. The practical deduction we would make from this is that no surgeon, however skilful and experienced, however preeminently gifted with the tactus eruditus, is entitled at once, and as if intuitively, to pronounce on the character of any tumour, and thereupon act incontinently; but ought in all cases to be patient, searching, and careful in his manipulations, neglecting to employ no legitimate means towards the obtaining of a true conception of the case. M. Lisfranc's deduction is somewhat similar, but not exactly; he urges the propriety of an almost indiscriminate use of the "exploratory trocar." Now we hold that this ought always to be the last means of diagnosis which is resorted to, and not until all others have been patiently and well tried, and yet proved unsatisfactory; and not even then, unless both patient and surgeon have made up their minds instantly to proceed with the extirpation of the tumour, if such it prove to be; otherwise much mischief is likely to result from the indulgence of what under the circumstances may be not inaptly termed an impertinent curiosity. All tumours, however simple originally, are apt to degenerate; and the ordinary cause of degeneracy is an excited action of their blood-vessels, which induces a sinister change in the character of their perverted nutrition. A blow; a casual inflammation in their vicinity; the absurd attempts to discuss them, which we formerly reprobated; habitual pressure, are all very likely causes; but none so likely as the perforation of their interior by an exploratory needle or trocar. We have more than once traced the untoward effects of this in the subsequent history of the case, and more especially in the section of the tumour after its removal at a remote period. From the hour of puncture the progress of the tumour was markedly accelerated, and all its characters rapidly changed for the worse; its section disclosed an originally fibrous or simply sarcomatous growth, fast becoming entirely encephaloid, and the nidus of this nefarious deposit was obviously in the track of the trocar or needle, as indicated by a bloody infiltration of the encephaloid substance, always a most ominous sign of present and future malignancy. Many surgeons evidently have no fear of any such casualty, and hesitate not to practise an exploratory thrust, as if it were a simple and innocent manipulation; boring tumours in search of pus, serum, or blood, as unconcernedly as an exciseman might probe a wain of hay in search of fluids more plainly contraband. According to our thinking, they thus, in nine cases out of ten, ensure a speedy and certain degeneration of the solid growth thus fruitlessly tapped; and we hold, therefore, that such exploration, as formerly stated, is only to be had recourse to in very doubtful cases, when other manipulations have failed to satisfy; and then only with the patient on the operating table, or prepared to ascend it at a moment's notice. 2. A tumour placed over the course of large nerves, blood-vessels, or other important organs, may seem to be completely separate from them; yet in many such cases the operator finds during his dissection that his previous examination has deceived him, the prolongations of the tumour often extending to a much greater depth than was externally indicated. Against such surprises, therefore, he should be upon his guard; forewarned he is forearmed. Several appropriate cases are adduced by M. Lisfranc in support of this axiom: among others that of a girl, who had a fatty tumour on the back of the neck, moveable and apparently superficial; in dissecting it out, however, M. Lisfanc had to expose the ligaments of the spine: no accident interrupted the recovery. 3. Some pendulous tumours of a narrow pedicle enlarge remarkably in their free portion, and cover a large extent of surface. In removing them, it is well first to amputate this pedicle on a level with the surrounding skin, as then the dissection for extirpation of the remainder can be conducted more easily and more accurately. 4. Moveable carcinomata of the breast may yet be deep and dangerous in removal; having become incorporated with the fibres of the pectoral muscle, and not gliding *on* these during manipulation, but *with* those on the subjacent tissues. It is only by very careful examination that such a condition of parts can be previously determined. 5. Pendulous heavy tumours sometimes by their own weight withdraw their deep attachments, which become more and more superficial. Art may sometimes assist nature in such retiring and commendable tendencies of the morbid formation. 6. Integumental incisions are much facilitated by previous tension of the skin; but when lines or points are important guides to the relative anatomy of the subjacent parts, while they are stretched, let them not be displaced; take for examples, the raphé of the perineum, and the lines indicating the site of the articulations. 7. As a general rule, the line of incision should be parallel to that of the subjacent muscular fibre; but to this there are exceptions. When important blood-vessels or nerves are concerned, we cut in the line of their course, and so run less risk of injuring them. In the forehead and face we often cut nearly transversely to the line of muscular fibre, finding it to be of more importance to be in the line of the habitual integumental folds, the result of muscular action. But such exceptions, as in other cases, only strengthen the general rule. 8. Incisions for the removal of tumours ought to commence where the principal blood-vessels and nerves enter, and advance steadily from that as a starting point. The nerves are cut at once, and thus the subsequent dissection becomes comparatively painless; the arterial trunk is compressed as soon as cut, and the operation in consequence is also comparatively bloodless. Following an opposite course, much blood is lost, an unnecessary number of ligatures may be required, and a severe amount of protracted pain is unwarrantably inflicted. In removal of the mamma for example, the elliptical incisions are commenced at the border of the axilla, and the dissection proceeds from that towards the sternum. 9. Free incision we formerly advocated in deligation of arterial trunks. We think equally well of the principle in extirpation of tumours; and therefore agree readily with M. Lisfranc in recommending its adoption, when not inconsistent with the safety of important neighbouring textures. 10. Deep walls of fat are inimical to adhesion of a wound. In operating on subjects of obesity, it is therefore advisable to remove a suitable portion of the subcutaneous fatty texture along with the tumour, by inclining the knife in an excavating posture. 11. In removing malignant tumours, especially the cancerous, we have two errors to avoid; too sparing, and too free removal of the integument. If over anxious to have an easily coaptating wound, we may spare skin and cellular texture already involved in the agency of the morbid product, and consequently certain soon to produce the disease in a worse form of malignancy. And if we remove a large extent of integument, the part will be prone to a return of the tumour, in consequence of the irritation caused by the tightness and puckering of the cicatrix; also experience shows adhesion to be more favorable to immunity than is tedious granulation. When in doubt, however, it is surely well to err on the safe side, and sacrifice everything to full and free removal of the diseased parts. 12. A large artery or nerve passing through a diseased formation may seem to be hopelessly incorporated with its structure. Yet if the tumour be not mali moris, the artery or nerve so situated is not to be rashly sacrificed in either the planning or execution of the operation. They are in a bad neighbourhood, badly employed, yet may pass innocently through without being at all implicated in the nefarious proceedings, as experience has again and again shown. A fine elastic cellular texture often permits the tumour to be extirpated wholly, without injuring arteries, veins, and nerves so circumstanced; gentle coaxing traction by the fingers, and pushing by the handle of the knife, ought to precede the use of the sharp edge in such dangerous localities. 13. During such gentle tearing and stretching of the cellular texture, however, be on your guard against the entrance of air, especially into veins; for experience has shown that such an accident is more likely to occur then, than during straightforward simple incision. 14. When it becomes necessary in a deep situation to divide a cellular prolongation of a simple tumour, in which a considerable arterial twig is placed, beware lest by its elasticity the bleeding point recede beyond your immediate reach, requiring a difficult and dangerous dissection for its subsequent security. Put in force the same precautions as in section of the cord during castration. Having already reprobated the system of digging into axillæ for glandular enlargements of a malignant character, *our* remarks are not directed to such cases. 15. When the case of a large and deep tumour involves a difficult and dangerous dissection, this may be facilitated by removal of the principal part of the growth already exposed; simple bisection of it may sometimes answer the same end. Should any considerable vessel or vessels, however, traverse the structure of the tumour, such procedure might prove both troublesome and dangerous. Partial emptying of the contents of a stout and large cyst may, on this principle, facilitate its removal when deeply situated, as at the angles of the jaw. The peritoneum is more easily separated from the abdominal parietes after opening of the abdominal cavity, than when this is entire. 16. When the knife has gone as deeply as seems consistent with safety, the remainder of the tumour may be attacked by ligature, that is, when the growth is of simple texture and innocuous tendency; as in the removal of central goitres, whose increase is seriously interfering with respiration.

*On the treatment of l'esthiomene* or *dartre rongeante*." 1. We have already reprobated empiricism in the treatment of burns and bruises, and would now do the like in regard to this complaint. Many and excellent remedies for it are frequently used too indiscriminately. Cauterization is the most powerful and most generally employed; yet will often not only fail to amend, but be sure to injure. The "liquid acid protonitrate of mercury" is preferred by M. Lisfranc, employed according to the following principles. 2. When acute and constant pain accompanies the disease, and redness of the sore and its neighbourhood is very marked, cauterization then employed will but add fuel to the fire. An excited vascular action is there, and must in the first instance be subdued by suitable means; and then, but not till then, cauterization may be expected to induce the most favorable results. Even then it may still be employed with undue severity, again lighting up the action which had just been extinguished; and therefore the first application should be especially cautious. Should over action, notwithstanding due precaution, result, the soothing treatment is once more to be had recourse to, instead of blindly persevering with the cauterization. In order to subdue the excited action, whether casual or the result of malapraxis, M. Lisfranc unhesitatingly advises general bleeding. We begin to suspect that he is much too fond of this (almost as bad as his countryman, M. Bouillaud, vide our No. XXVI, p. 405); and would rather content ourselves with local bloodletting by punctures or leeches; agreeing, however, very fully with our author in the propriety of placing the latter at some distance from the actual disease, otherwise there is much probability of the wounds degenerating. The general bleeding he employs is revulsive, not spoliative; practised as far as possible from the seat of the disorder, not exceeding three or four ounces in amount, and sometimes repeated pretty frequently. 3. As to repetition of the cauterization, M. Lisfranc justly censures the custom of defining such matters by a fixed period. "Let it be repeated every eight days," say some, with surprising exactitude. This may be all very well with a shower-bath, a purge, or a diaphoretic, but is not so suitable to the cautery, actual or potential. We must watch effects, and be guided by circumstances. If after the first application, the sore cleans and then advances favorably by healthy granulation, why repeat the cauterization at all? unless indeed you wish to excite thus artificially a disease similar to, if not worse, than nature's product, which just before you had been in the way of curing, but which you now seem wishful to reinduce. And on the other hand, when the sore, at first a little amended, rapidly reverts to its former perverted condition, why wait for the expiry of any definite period? Attack it immediately. 4. Before applying the liquid, the ulcerated surface is to be carefully wiped and dried, otherwise the fluids of the sore combine rapidly with the caustic, forming a white coating under which the diseased surface lies intact. 5. The part is touched lightly with the caustic; the object is not to disorganize it wholly, but rather to change its action in some mysterious way, designated by M. Lisfranc "a modification of the vitality of the tissues;" the term "cauterization" therefore is rather a misnomer here. 6. When the diseased surface is extensive, it ought not to be all touched at once, but by instalments; and often the distant parts are, as if by the example of the others, brought into a better mode of action before their own turn for being touched has arrived; thus saving both time and pain in the cure. 7. Such is our author's local treatment. In our own experience, we can speak favorably of both the "liquid protonitrate of mercury" and of the plain nitric acid; the principal fault of the former is the long persistence of severe pain after its application; but we have long since come to the conclusion that fully as much is to be done by internal or constitutional treatment, as by remedies directed to the part alone. Arsenic, iodine, hydriodate of potass, sarsaparilla, and in some very rare cases mercurial preparations, are valuable, each in its own place and way, after rectification of the primæ viæ, as well and as fully as lies in our power. In general, M. Lisfranc believes that excitant remedies are much too indiscriminately employed, not only to the retardation of the cure, but often to the aggravation of the malady. From this he would carry us back to his favorite system of revulsive general bleedings; the principle is good, the practice so-so.

In a short chapter, M. Lisfranc well insists on the important difference between *congestion* and *inflammation*, both as to their nature and treat ment. Some people have a constant vision of inflammation before them, are haunted by it, and become perfect Sangrados in practice, to the sad detriment of their patients, themselves, and their profession. But this subject is surely by this time sufficiently distinct in our own medical literature, and requires no illustration here. In the same chapter M. Lisfranc justly reprobates the protracted use of poultices in wounds which have been inflamed. So long as inflammation actually exists, they are useful towards its subsidence, if not heavy or rancid; but after a time the wound becomes stationary, is somewhat painful, has lips swollen and red, emits a profusion of purulent secretion, and makes no progress towards reparation. Omit the poultice, employ a simple detergent and protective application, and a rapid amendment will immediately ensue. The poultice was here to blame; but not by its "relaxing" effects, as usually stated. We believe that it induces and maintains a *congestion* in the part, on which congestion all the above symptoms and appearances depend; and to remove that congestion all that is necessary is to remove its cause—the poultice. Poulticing of the chest is a favorite remedy with some for inflammatory affections of the interior; its modus operandi is by obtaining sanguineous flow to the surface; and this seems a key whereby we may explain its unfavorable effect when too long used as a direct application to wounds. And, by the bye, this is another example of the superiority of water dressing over the poultice as a vulnerary, as formerly stated; the former having all the good qualities of the latter, and being less likely to injure by negligent or empirical use.

*On hernia.* In regard to the treatment of this most important disease, there seems to be two strongly adverse opinions at present dividing the surgical world. The one, that when strangulation has been fairly established, we cannot operate too soon; the other, that the operation is to be long deferred; or rather that by the taxis and subsidiary means, judiciously and patiently persevered in, operative interference will in most cases be rendered wholly unnecessary. Here we find the old adage coming true once more, "in medio tutissimus." And the sensible man will not attach himself to either extreme, be it *gauche* or *droit*, but wisely steer a middle and a natural course: being satisfied by sober experience, that urgent cases will not unfrequently present themselves, in which the knife's edge must not be withheld for one half hour; while others, and perhaps they may constitute the majority, not only warrant but call for delay in operating, and may eventually yield, in all safety, to the taxis. M. Lisfranc's opinion on this subject seems scarcely to occupy the *juste milieu* which we have endeavoured to indicate, but rather inclines to the non-operating extreme, nearly to the same extent as his countryman Malgaigne. We are also aware that in this country, a living surgeon of some local celebrity has long boasted that he has as yet met with no hernial protrusion, strangulated, incarcerated, or free, which has been able to resist the stern and rude compulsion of his finger's gripe. But we have not yet learnt how many cases of death by gangrene and fecal effusion have occurred in this gentleman's practice. It would require a power of eloquence even more persuasive than that of his manual organs to convince us that such dire casualties have been either "few or far between." We shall briefly advert to the more interesting and important of M. Lisfranc's observations on this subject. 1. Two questions are asked: In strangulated hernia, are a great number cured who have been operated on immediately? Are many strangulated herniæ simply reduced without untoward results? M. Lisfranc seems to think that to both we cannot reply, Yes; but we differ from him in this, and believe the true answer to be in both affirmative: at all events, we see no reason why this should not be. Statistics afforded by M. Malgaigne are adduced to show that a negative is the due response to the former of the interrogatories; but these statistics, at least, as quoted by our author, are glaringly incomplete, and therefore totally irrelevant to the point at issue. The mere naked statement that out of 183 operations there resulted 114 deaths does not militate against the operation itself, unless it be at the same time shown, as is not attempted, that these cases were operated on at an early period, and otherwise under favorable circumstances. Nor because we are merely told that, between the ages of fifty and eighty, ninety-seven operations produced seventy deaths, are we to believe that therefore Boyer was wrong in saying that in old people the performance of the operation ought to be especially early. In these fatal cases, was the operation timeously and well done, or only at the eleventh hour, and with the rashness of despair? If the former, then Boyer was wrong; but if the latter, his doctrine is still unshaken; and before our opinion can be made up on such data, the preceding interrogatories must be plainly and fully answered. 2. M. Lisfranc comes closer to the point when he tells us that he has himself operated, and seen others operate "in a great number" (his footrule exactitude would be of use here) of cases, where "inflammatory strangulation had existed only for some hours,"—and yet the operation seemed to be very murderous. Such is his experience; that of others differs. Besides, though we must award to his operations the meed of dexterous performance, yet it is not shown that they were even in these cases timeously had recourse to. For every practical surgeon knows that an "*inflammatory* strangulation" may in less time than is occupied by "some hours" render all hope from any operation, however skilfully executed, wholly abortive. On behalf of the accused operation, then, we beg to plead "not proven." 3. We are told that Jean Louis Petit met with little success, but sad reverses, in his operations on non-strangulated herniæ with the view of obtaining a radical cure. Jean Louis Petit deserved no better fate in such a shameful business. Operations of "complaisance," and these were scarcely so good, seldom have a prosperous issue. Because a man has his toe amputated, on account of a corn, in order to improve the look of his boot, and has both limb and life endangered by a subsequent erysipelas, that is no reason why a similar operation should not be performed when the said member has been crushed to a jelly by accident. And though amputation of an anchylosed knee, on the score of mere deformity, has not unfrequently had a fatal issue, is this to debar us from performing a precisely similar operation on account of hopeless disease of the articulation? We trow not. 4. M. Lisfranc has "performed the taxis in many cases with perfect success." So has every experienced surgeon; and what is more, so does every experienced surgeon expect to do again. But such facts by no means liberate him from the fear, that not a few cases may occur, in which he must desist from the taxis, even at an early period, and take up his bistoury. "There is a time for everything," a truth which practical surgery teaches to many with less quickness of apprehension than a Solomon. 5. It is usually held that abortive attempts at reduction, when long continued, aggravate the disease. M. Lisfranc denies this, when the operation is immediately performed on cessation of such attempts. He forgets that the cause of inflammation in an important texture, whose power to control increased and perverted action is impaired, has been for some time in active operation; and that the mere cessation of that untoward exciting agency may not be sufficient to extinguish the action already kindled, and prevent its progress to a deleterious issue. Surely there is no sounder maxim in surgery than that in those cases in which a prima facie suspicion is given of a necessity for operation being about to occur, forcible manipulation ought not to be employed for any lengthened period; and, more especially when the strangulation is already of the inflammatory character, otherwise the prospects of the case must be clouded darkly indeed. 6. Moreover, M. Lisfranc makes light of the chance of returning a gangrenous intestine by heroic taxis; congratulating himself that there are so few such accidents on record. But ought there to be any? And are not such cases, however few, proof demonstrative to what fearful disasters the taxis may lead, when blindly and brutally persevered with? He contends that taxis is the rule, and operation the exception. We admit this; but at the same time are fully convinced that *many* exceptions must ever occur to the general rule. We by no means undervalue taxis, but believe that like every other good thing, it not only may be but is frequently abused; to be successful, indeed not to be injurious, it must not only be well timed, but judiciously and carefully conducted. 7. M. Lisfranc admits that there are cases in which the taxis is not to be attempted. When the symptoms of strangulation are followed suddenly by a perfect calm, deceitful to the patient and his friends, most alarming to the surgeon; the intestine is already gangrenous, and if now reduced, loss of life must follow. It requires no witchcraft to tell that in such cases the taxis is inexpedient. But we hold that the period at which the taxis has ceased to be hopeful of benefit is much earlier. When the inflammation is marked and progressive, and the tumour tense, tender, and unyielding, the pulse abdominal, and the system plainly getting worsted in the struggle, then it may be hours before M. Lisfranc's advanced class of symptoms have come on, then is the time to cease manipulation and haste to operate, with the double hope of safety of life both to the patient and to the important texture involved in the disease. 8. Further, it is admitted by our author, that there are certain cases, "happily rare," in which an insidious inflammation is slowly advancing to gangrene, and yet without any adequate manifestation of such a mischief; and in which an indiscriminate taxis would doubtless be prone to return an intestine either sphacelated or about to be so. Two such accidents happened to M. Lisfranc himself; and therefore he comes round so far as to admit that after the fourth day of strangulation the taxis ought not to be attempted, (ought a fourth day of strangulation ever to arrive?) And he also announces that when a narrow-necked hernial tumour is tense, with an evidently grave inflammation raging in the strangulated parts, the taxis is not only useless but highly dangerous, and therefore not to be attempted. 9. In regard to artificial anus, M. Lisfranc agrees with M. Dupuytren in the propriety of delay in the use of the enterotome; because the aperture may heal either spontaneously or by the influence of pressure alone; and because premature application of the instrument may rupture recent adhesions, producing an acute inflammation which may seize on the peritoneum, and end fatally. M. Velpeau applied the enterotome on the sixteenth day; death resulted; and M. Lisfranc does not fail to tell him of his mistake. But we must vindicate M. Velpeau's opinions on this topic, at all events as held "at the present writing." In our Twenty-fourth Number, p. 460, he will be found detailing the dangers attending the unguarded use of the instrument—peritonitis; fecal extravasation by ulceration, or by premature breaking up of limited adhesion, and no formation of compensating deposit; also inclusion of a portion of sound bone in the embrace of the blades. 10. In applying the taxis, M. Lisfranc agrees with the majority of surgeons in this country in the propriety of ascertaining the exact line of the hernial neck, and transmitting the reducing power in that direction. 11. There seems little doubt but local bloodletting ought to be more frequently and fully practised in inflamed and strangulated hernia, more especially if the inflammation have preceded the strangulation, and otherwise may reasonably be considered its cause; but M. Lisfranc justly reprobates the direct application of leeches to the tumour itself: sensibility of the skin is increased; the taxis becomes more painful, the patient more unsteady; the blood makes the surface slippery, rendering the manipulation both difficult and ineffective; ecchymosis masks the subintegumental textures, and complicates incisions when ultimately had recourse to. The leeches will be equally effectual if applied in the *neighbourhood* of the tumour. 12. M. Lisfranc disapproves of relaxation of the abdominal parietes during attempts at reduction. He believes that when relaxed, they apply themselves to the contained viscera, diminishing the abdominal cavity, and intimating that there is no room for the excluded portion; that they are apt to yield too much to the taxis, sliding under the fingers; that as tense parietes favour passage of the abdominal contents, in one direction, they will also do so in the opposite; and as relaxation of them is unfavorable to protrusion, it will be equally so to reduction; the fist is easily pushed through a hole in a towel or blanket which is stretched, while one that is loose may foil many efforts. By such reasoning as the above, M. Lisfranc is led to recommend moderate tension of the abdominal parietes during the application of the taxis. We are not prepared to coincide with him, especially in the justness of his similitude regarding the hole in the blanket; but see no reason why, failing in reduction during abdominal relaxation, we may not make new attempts during moderate tension. In crural hernia, it seems very plain that much benefit is to be invariably expected from simultaneous relaxation of both thigh and abdomen. 13. In determining the direction of the reducing power, M. Lisfranc again pulls out his foot-rule, and wishes to fix us down to certain invariable centimetres and millimetres. Here we are wholly in favour of "a sliding scale," knowing well, that according to the age, size, and other circumstances of a hernia, the form and direction of the abdominal apertures and canals must frequently change; and that therefore such fixed measurements are more likely to be fraught with error than with benefit, especially in the hands of the inexperienced surgeon, for whom they seem chiefly intended. 14. Tobacco enemata are held by M. Lisfranc to be extremely dangerous in this disease, and not expedient as auxiliary to the taxis. Agreed. 15. In employing the taxis, in favorable cases, M. Lisfranc enjoins steadiness and perseverance in the efforts, taking a hint on this subject from nature's means of pushing the child's head along the vagina. Again we concur, but under the limitations formerly specified. 16. As to the use of purgatives after reduction, M. Lisfranc coincides with the general opinion as to their evil tendencies when inflammation exists; but seems inclined to recommend them otherwise. To this we demur. It has been satisfactorily shown by Travers and others, that a temporary paralysis of the constricted bowel occurs in most cases, when the strangulation has been severe, and persists for some considerable time after reduction. Purgatives during this condition must be highly dangerous, stimulating a part which has not yet recovered the power of obeying that stimulus; and is most likely, besides, to induce an excited vascular action, where there is little power to control it; and when, therefore, gangrene is more than probable. For "purgatives" read "laxatives" in the treatment of recently reduced herniæ. 17. When inflammatory symptoms follow reduction, M. Lisfranc considers the external application of mercurial ointment to be highly efficacious, according to the formula of M. Serres d'Uzés. Two pounds of the ointment are used in the course of forty-eight hours, spread on the lateral and anterior parts of the abdomen, laid on layer by layer every two hours, until the whole dose is expended. If two days' use of this, according to M. Serres, prove insufficient, the method is to be abandoned in favour of others. But M. Lisfranc has a higher opinion of it, and states that he has used it successfully, to the extent of ten pounds in five days, and sixteen pounds in eight days!

We are rather disposed to think this a clumsy method of exhibiting mercury, and would rather undertake to *pound* the peritonitis by its internal administration, leaving the abdominal surface free to be more usefully and energetically handled by depletion or counter-irritation, according to circumstances. 18. Cold is not in favour with M. Lisfranc as an auxiliary to the taxis. We agree with him that intense and continued cold, as by ice, is most dangerous in urgent inflamed strangulation, and is more likely to promote sphacelus than reduction. But in the more chronic yet equally obstinate cases of strangulation, without marked inflammation of the extruded parts, we have seen the most obvious benefit from the use of cold by evaporation ; nitrous æther, for example, applied to the tumour, and its evaporation promoted by sufflation. In two cases lately, one of inguinal, the other of crural, both very large, we had used the taxis as powerfully and as long as prudence seemed to warrant, yet without making the slighest impression on the tumour. An interval occurred, during preparation for the operation, and that was occupied in the use of the evaporation, scarcely with the hope of benefit; yet after its brief employment, a very slight manipulation, in one case by the patient himself, sufficed for satisfactory reduction. We are not prepared to explain fully the modus operandi of this remedy, but believe that it chiefly acts by condensing the gaseous contents of the bowel, thereby effectually diminishing the tension and bulk of the tumour. At all events, in whatever way it acts, we are fully convinced by experience, (and, as M. Lisfranc often tells us, "there is no resisting the brutality of facts,") that it is in many cases a most powerful auxiliary to the taxis; and should be sorry to operate on an uninflamed strangulation without having tried its previous employment. In an inflamed hernia, its use must be highly prejudicial for very obvious reasons. 19. Sundry examples are given of varieties in the site of stricture. A case by Lecat, for example, in which the bowel, after having perforated the anterior part of the sac and the posterior part of the tunica vaginalis, became strangulated by the margins of this unusual opening. The expediency is also well insisted on of inquiring carefully into the previous history of the case, to ascertain whether or not the tumour in its ordinary state had been wholly reducible; otherwise useless attempts at taxis may be hopelessly persevered in, or else a portion of reducible bowel left unreduced. 20. In crural hernia, we are not to suppose that the neck of the tumour is limited to the passage through the abdominal parietes; the canal of its transit extends to the fascial opening for the union of the femoral and saphena veins, over which it turns resting its fundus on the abdominal walls. M. Lisfranc rather ridicules the ordinary mode of undoing this turn of the tumour, bringing it into a line with its neck, before applying the taxis. But here we think he is wrong, and contravenes one of his own general principles formerly noticed. 21. The usual posture of the surgeon in reducing hernia is beneath the site of protrusion, pushing from him. M. Lisfranc's experience leads him to prefer an opposite mode of procedure, standing above and drawing the tumour towards him; but still he does not wish this to supersede the ordinary mode in every instance. Many important practical points relating to the treatment of hernia are not even touched on by M. Lisfranc; but we cannot be expected here to supply the deficiency.

*If fistula can produce the indurations which surround them, often*

*such indurations are the means of maintaining the fistula.* In treating an ulcer of the leg complicated with callosities, cicatrization is scarcely to be obtained without previously bringing the indurated tissues back to their normal texture; and it is the same with fistulæ. To reduce the indurations rest, leeching, pressure, and preparations of iodine are the means employed. Still the fistula may not heal; then injection is recommended, and for this purpose M. Lisfranc again brings forward his favorite chloride of soda, proportioned in strength to the exigencies of the case. Failing injection, pressure: failing all, incision. And always opposing "an obstinacy of treatment proportioned to that of the disease."

*Wounds made in "tissus lardacés non squirrheux," and not arrived at the stage of softening, may be the means of restoring them to a healthy condition.* This principle M. Lisfranc wishes to apply to the saving of limb and life in amputations; showing that in the leg, for example, we may amputate there on account of carious bone, though placing our incisions in diseased soft parts; and are not *compelled* to operate on the thigh. And in similar affections of the fingers or metacarpal bones, the valuable organ of prehension may oftentimes be comparatively little mutilated, by cutting in and not above the soft indurated parts. The loss of blood and the subsequent salutary amount of inflammation have the power of restoring these to a normal state; the amendment seeming to commence on the surface of the wound, and thence gradually to extend outward. In these circumstances, however, the flaps must be made unusually full, otherwise subsequent atrophy of them will leave but a sorry covering to the stump. And during the early period of treatment we must be especially careful to avoid an excess of inflammatory action, which in such textures would be certain to advance hastily to the most unfavorable results. At first the flaps are not approximated, but left free to suppurate and amend. When, however, the expected improvement has taken place—as evidenced by subsidence of swelling, diminution of discharge, and appearance of healthy granulation—then the surfaces are brought and retained in contact, with good prospect of rapid and satisfactory union.

On the same principle M. Lisfranc proposes to treat the indurations by which ulcers (indolent) are so frequently surrounded, by scarification, a method much extolled by Ambrose Paré, and which, our author complains, has most unjustly been permitted to fall into desuetude. We suspect, however, that the treatment by traction and pressure, as first proposed by Mr. Baynton, and since modified and improved by various practitioners in this country, will be found adequate to the accomplishment of the same indications at a less cost of time, pain, and risk. To make "forty or fifty scarifications" of an ulcerated limb "every eight or ten days" is scarcely consistent with the usual tenor of British surgery. But as M. Lisfranc does not enjoin such heroics "until other means have failed," we are happy to feel that we need not anticipate being often, if ever, compelled to employ them.

*General rules regarding the disarticulations.* In this chapter our author lays down precise rules for guidance in the manipulations, founding all on his favorite "échafaudage des linges." But into such elementary paths we are not expected to follow him—much to our relief, to say the truth; for the task would require the oft-repeated use not only of the foot-rule and compasses, but of various philosophical implements necessary for ascertaining angles with mathematical exactitude. This system, according to our author, has been most potent in quickening the march of surgery, "thanks to his firmness and courage;" but on this point we are disposed to be sadly sceptical, for reasons formerly given in, and to which we wish to be understood as still adhering.

*Of the simple atonic ulcer.* Under the denomination of "atonic" M. Lisfranc plainly includes the "weak" and "indolent" ulcers of other classifications. A few of his opinions on this subject require notice. 1. He supposes that it is not mere atony or debility that induces such morbid formations so frequently in the lower extremities, else they should be oftener on the toes and foot than on the leg. Retardation and obstruction of the venous circulation is obviously more intimately connected with their origin. In the case of varicose ulcer, every one admits the varix to be the principal cause of the breach of continuity; to the simple ulcer M. Lisfranc wishes to allocate a similar though less marked connexion, as to origin, with venous congestion. With this we wholly agree. 2. But when shortly afterwards we find him stating that this venous congestion operates thus ulceratively "by a gangrenous inflammation, *sui generis*," we find fault with his terms, and differ with him *in toto.* The venous congestion predisposes to inflammation; a slight exciting cause suffices to kindle the flame; by the venous congestion the healthy functions of the part have previously been materially interfered with, and its *powers* consequently impaired; it controls the excited action badly, if at all, and falls before it an easy victim, by the disintegrating process of ulceration. At first the ulcer is an inflamed one; but by and bye such characters leave it, and it *ought* to become a simple, healthy, purulent sore, evincing the justice of its claim to this honorable appellation, by pushing forward healthy granulations, and showing an evident wish to cicatrize laudably. But the cause which rendered the attempts to control the inflammation abortive is still in operation, rendering equally nugatory all efforts at reparation; and thus the sore very soon dwindles down into the "atonic" ulcer. And thus, by the ordinary causes of disease is the sore produced; not by inflammation, "gangrenous" or "sui generis." Such terms thus employed are loose, meaningless, at variance with sound surgical pathology, and obviously tend to foster empiricism—a leprous excrescence on our art which M. Lisfranc is in general the loudest to condemn. Ulcers of the lower limbs are often admitted into hospitals in a sloughing or gangrenous condition; but that is a casualty easily accounted for: action beyond power; and moreover such an ulcer is not then to be termed "atonic," but inflamed. 3. Is M. Lisfranc, quoting from M. Gensoul, in earnest when he contemplates ligature of the femoral artery as a cure for ulcer of the leg? We hope not. Did it succeed, it might doubtless be a point in favour of his view of the origin of the malady; but in the case of failure we fear that, in this country at least, the legal code of the realm would come to the assistance of the offended laws of humanity. 4. The reasons why ulcers are situated more frequently on the left lower extremity than on the right, are well stated. The sigmoid flexure of the colon, often full, passes over the left external iliac vein, while on the right side this vessel is free. The left iliac vein is covered by the two common iliac arteries; again the right is comparatively free. These arteries interfere with the venous current not only by virtue of their mere weight, but also by virtue of the column in them being more impetuous and powerful than the counterflow in the vein. 5. That either leg should be the frequent seat of ulceration, preceded and induced by venous congestion, is readily understood by reflection on the fact, first shown by M. Serres, that the saphena vein is unprovided with valves, from the inner ankle to the lower part of the calf. In many subjects, from the knee to the foot there are no valves at all in that vein; and when present there are seldom more than two—one at the lower part of the calf, the other near the inner condyle of the tibia. 6. In the treatment of weak ulcers of the lower limbs, so far as we are aware, due prominence is awarded by all good surgeons to the indication of obviating as much as possible the venous remora, by bandaging, attention to position, confinement to the recumbent posture, &c. But M. Lisfranc is heroic on this topic, and attacks the evil more boldly. By operative interference he obtains, or wishes to obtain, obliteration of all the superficial veins in fault, driving the current to a deeper channel; performing, in fact, the operation which others only deem warrantable in the most troublesome and obstinate cases of varix. The theory on which this mode of treatment is founded is faultless; but possessing as we do a most cowardly yet salutary dread of phlebitis, and believing such interference with veins to come nearly, if not wholly, under the denomination of the "opérations de complaisance"—the unfortunate results of which are proverbial—we must be excused from either following or sanctioning the practice, until guaranteed by further experience that disaster is not likely to outweigh the anticipated benefit. 5. M. Lisfranc admits that phlebitis is by no means an unlikely result of his practice, but congratulates himself that *as yet* he has lost no patient from this cause. The principal means in which he trusts for its subjugation is the application of leeches between the inflamed point and the heart; a very good principle, breaking the stream of flame, as firemen in desperate cases direct their aqueous shower, not on the doomed building, but on its neighbour as yet intact. 6. M. Lisfranc speaks of the varicose ulcer as a distinct species. Are not all kinds of ulcer found complicated with varicose veins in the same locality? 7. From the "varicose ulcer" he has known fatal hemorrhage occur; and in the dissection of such cases he has found the saphena much thickened in its coats, and presenting an open section like that of an artery, an inert tube through which the blood flowed freely and uncontrolled. 8. Absorption is more sluggish in an ulcer than in a recently-exposed surface; on the former morphia may be almost inert, whilst on a blistered part much activity of operation is to be expected. On the extreme surface of the ulcer is a new formation not yet fully endowed with all vital properties, and absorption, amongst the rest, is deficient. On this account the calamitous reabsorption of pus by casualty or improper treatment is less likely to occur in the case of an old ulcer than in recent suppurating wounds. 9. On the practical question as to the expediency of drying up and healing old sores, we have the following sensible remark: "Examine the state of the viscera, especially in the chest and abdomen; if they are sound, if the general health and system seem good, if the ulcer in its formation have not dispersed another and more important disease, cicatrization is to be attempted," and "vice versâ;" in extreme cases never omitting the precaution of establishing an issue conveniently in the vicinity, as an atonement for the ulcerous discharge to which the system has been long accustomed. Should evil nevertheless threaten, the original sore ought doubtless to be reestablished with all convenient speed. 10. When a sore proves sluggish in healing M. Lisfranc "immediately has recourse to stimulants." We dislike the phrase "sur le champ" here; for sudden change from bland to stimulating, in the applications to sores, is one of the worst errors in every-day surgery; the transition ought uniformly to be gradual, unless in the sloughing or phagedenic, when indeed arrestive measures must be prompt as well as powerful. 11. Ointments are held by M. Lisfranc to be "often of great utility." In this country the lotions have nearly exterminated the unguents, and, we think, most righteously. 12. We believe that healthy pus is not only bland in its nature, but that it is secreted on a granulating surface for the express purpose of protecting the tender granulations from atmospheric influence and other sources of injury, until they are finally covered in and protected by the new cuticular membrane. And we consequently hold the wiping, sponging, and washing of sores, especially when oft repeated, to be vile, meddlesome, mischievous surgery. M. Lisfranc espouses an opposite doctrine, and falls into the old mistake of supposing that the *pressure* of confined and accumulating pus, producing serious mischief in hard and soft tissues with which it is in contact, is proof positive of an *eating* and *acrid* propensity in the fluid itself. Frequent dressing and careful removal of pus constitute his rule; the opposite the exception. And the more irritable the sore, the more diligent is he in wiping away the pus suspected of tending to increase the irritation. Wanted the means of converting a sound, healthy sore into either an irritable or an inflamed one, M. Lisfranc has solved the problem—unwittingly. There are extremes in all things; and we grant that dressings allowed to remain for days unchanged will, by putrefaction, render the puriform fluid most plainly irritant to the surface with which it is in contact, and, moreover, further noxious to the patient and his neighbours, by tainting and deteriorating the atmosphere; indeed, we think we have seen the occurrence of hospital sore attributable to this cause; but, as a general rule, the less a healing sore is interfered with the better; and certainly when it is dressed, merely the superabundant pus should be wiped away, not from the sore itself, but from its vicinity. M. Lisfranc labours to support his opinion by argument, but fails signally; in one point he palpably contradicts himself: in atonic ulcers he thinks it a good plan to dress the sores seldom, that they may have the full benefit of the rancorous tendencies of their own secreted pus; and in the very next sentence he advises us, "when the vital properties (of the sore) languish, (to) wipe the denuded surface scrupulously." (p. 527.) But on this subject we need not dwell; satisfied that the influence of M. Lisfranc will not be sufficient to break up the juster notions now held regarding the dressing of sores in this country. 13. In obstinate, indolent ulcers we are again recommended to attack the surrounding callosities by incisions. We cannot help thinking that most people, especially out of the profession, will be reminded by this of the old adage bearing on the impolicy of manufacturing ten holes in the attempt to tinker one, and will prefer, along with us, the treatment by traction, pressure, and stimulation; the more especially as in the next page (529) we are told by M. Lisfranc that it is quite possible such incisions "may occasion purulent reabsorption, and compromise the patient's life." 14. Some sores of the atonic class resist even M. Lisfranc. By two elliptical incisions they are dissected out, "as if a tumour;" and "thus a very rebellious ulcer is converted into a recent wound, which heals readily." We have not yet encountered an ulcer of any of the simple classes warranting such a summary ablation; it is possible a more inveterate species is to be found in Paris. 15. The solution of the chloride of soda is extolled as a stimulant application to sores, and, we doubt not, with perfect justice. The best of such lotions, however, will fail after a time, and it is only by a judicious alternation that a continuously favorable condition is to be maintained. 16. For repressing exuberant granulations M. Lisfranc seems to employ cauterizing substances alone. Dry pressure by compresses of lint, judiciously applied and retained, are, in our opinion, equally effectual, and less likely by accident to prove injurious. 17. In dull, obstinate, sores, whose surface has assumed a mucous character, the "liquid protonitrate of mercury" is recommended; this remedy is coming into use in this country, and, we doubt not, will be found available in effecting a "change of action" in many sores. As an enemy of phagedæna we have already attested its prowess. 18. It is often no easy task to heal a sore; and yet a more difficult one remains to keep it whole. Uniform support is a principal means towards this desirable end, and is usually effected by means of a laced stocking, which ought not to be *uniformly tight*, strictly speaking, but slacker above than below, in order to favour the venous return; the preferable texture for its construction is dogskin, according to our author; the upper part may be made of caoutchouc; and, to prevent its slippping, it may be attached to the drawers. If the patient's circumstances permit, obviously he ought to keep the limb at rest for some considerable time after cicatrization; it being well understood that the new textures are at first feeble and imperfectly organized; yet each day adds to their power and organization. 19. M. Lisfranc enters on a review of the various modes whereby veins may be obliterated; holding that, "if an ulcer have resisted all therapeutic means, if, in other words, it is impossible to heal it, if it condemns the patient to absolute repose, if it place his life in danger, if it renders amputation necessary, the vein ought to be attacked." Not, however, in old people of unsound viscera, not when the veins are already in a state of excitement, and not upon a dilated or varicose point of the vessel. A healthy part above the collateral veins is the point of election. Subcutaneous section, according to the method of M. Guerin, is favorably considered by our author; but his experience on this matter is as yet but limited; he has performed the operation once, and with success. Formerly he was in the habit of cutting out two inches of the vein—rather a clumsy operation, as M. Velpeau told him; and from this gentleman's charge to that effect he is scarcely extricated by a bitter reclamation (p. 544 et seq.) And here we would take the liberty of hinting to our author, in a friendly way, that M. Velpeau is not so bad a surgeon as M. Lisfranc—takes him to be. On the whole, the proceedings most favorable for obtaining obliteration of the veins are,

(1) those of Messrs. Franc and Ricord; (2) the subcutaneous section; and (3) resection of the vein, by his own operation just alluded to. In this country practitioners have almost unanimously selected No. 1; the twisted suture placed beneath the vein, and retained for a period varying according to circumstances; and as yet, so far as we are aware, they have had no reason to repent them of their choice. 20. M. Lisfranc does not admit ever having caused death by operations on veins, but his patients have died "from other causes," and he has dissected them. He has found the saphena obliterated, resembling the umbilical vein in the adult, from the point of operation down to the big toe; the collateral branches participated largely in the same condition: no traces of the varices remained, and obviously the circulation had been chiefly carried on by the deep venous system. Hence he infers that relapse after such operations is extremely improbable. We wish he were right.

*On white swellings of the joints.* This is the longest chapter in the book, and the least satisfactory. At the outset, we quarrel with the term "white swelling." It has been for some time dismissed in disgrace from the pathological vocabulary of British surgery, as vague, unmeaning, unscientific, and pregnant with tendencies to practical error, or at all events to blind empiricism. At one time, with us, everything in the shape of disease affecting the joint was dubbed "white swelling," and forthwith treated according to that name merely, not according to symptoms and circumstances. From such a misfortune we have been delivered: but M. Lisfranc, unfortunately, is still in the thrall of bondage. His *improved* definition of the term adopted from a M. E. Margot, would really warrant a treatise "de rebus omnibus (concerning joints) et quibusdam aliis." But bad as is the beginning, and commonplace and elementary as is the major part of the chapter, yet we shall find certain portions by no means barren of interest. 1. One word more as to the odious term. In order to show that he is not in the error of supposing all "white swellings" to be non-inflammatory, he proposes to divide them into "white swellings" and "red swellings." This is a little better; and as an omen of further and more healthy improvement, we are thankful for it. "Black spirits and white, red spirits and gray," are better than all white; but there is still too much of the "mingle, mingle, mingle." 2. Among some operating surgeons in busy practice, there is to be found a degree of recklessness as to amputation of diseased joints; looking too intently to the local disease alone, and not taking into consideration the fitness and working of the rest of the system. On this point our author is most sound, exhorting us as often as we are consulted in a case of "white swelling" to examine with scrupulous attention the state of the abdominal and thoracic viscera. If they are diseased, the joint affection may be an effort of nature for their relief, and must not be rashly interfered with. As it cures they may deteriorate; they are at first to be amended, if possible. And before operating in such cases, especially when suspicion of tubercular cachexy is urgent, a prognosis not based on a careful and accurate examination of the internal organs is worse than valueless. Chirurgery is not applicable here: in medico-chirurgery alone is there safety. 3. We are glad to find M. Lisfranc decided as to the paramount importance of *rest* in the treatment of joint affections. We honestly believe that it is in consequence of better means and more honest endeavours to obtain this indication, that

LIBRARY OF THE

Orleans Parish Medical Society the joint-mending of the present time is so strikingly superior to all that is foregone. We are very confident that no one not impressed with this idea will ever meet with credit or success in the practice of this department of his profession. And we are rather inclined to think that even M. Lisfranc, admitting the principle for which we contend, allows too many and too great deviations from it in his practical inferences; especially in regard to those of his "swellings" which are, par excellence, "white." 4. Abscesses formed *around* the "white swelling" we are advised to open as early as possible, for fear of evil consequences by delay. But abscesses *in* the "white swellings" are considered by M. Lisfranc as highly useful and commendable formations; and he accordingly enjoins us to open them as late as possible—the *devouring* pus, by remaining in contact with the "indurations," proving a most powerful discutient. (p. 582.) This runs counter to all our notions of sound surgery. Had we merely superficial "indurations" in the neighbourhood of common textures which could easily enough be spared, we should not grumble. But the destructive element is in juxta-position with one of the most important structures of the frame, and may at any time and with little or no warning leave its legitimate repast—the common textures—and prey fatally on that which we are most anxious to save. Out of his own mouth do we convict our author of error here. For in the very next page a case is related of fatal suppuration of the knee-joint, the occurrence of which any one but M. Lisfranc would have little hesitation in attributing to important delay in the opening of an abscess in the neighbourhood. 5. Leeches, applied to the number of three, four, six, and even eight, and the bleeding permitted for about a quarter of an hour after their separation, are held by M. Lisfranc to be stimulating, by determining blood to the part leeched, on the principle formerly explained. This is important, and ought never to be forgotten in practice. If the local depletion be intended to be spoliative and, consequently, sedative, it must be in greater quantity. M. Lisfranc supposes the minute leeching to be useful, as first excitant and then absorbent in the non-inflammatory swellings, and employs it accordingly. Repetition, however, he admits, is not unlikely to induce an erysipelas. 6. Compression has long been deservedly a favorite means of treatment in the chronic and indolent affections of the articulations; retarding the influx of arterial blood into the affected part, and thereby opposing perverted nutrition, stimulating its power of absorption, and effectually obtaining the precious immobility. But by indiscriminate use this is liable to abuse; and M. Lisfranc justly asks, "If the use of opium, antimony, &c. be regulated by doses, why not *dose* pressure also?" One uniform amount and mode and duration of pressure is not to be enforced in all cases, but it ought to be regulated entirely by the peculiar circumstances, past, present, and future of each individual case. And, in general, the older, the harder, the more indolent and dull the swelling, the more energetic the form of pressure to be employed. M. Lisfranc divides the remedy into five degrees, varying from slight pressure by means of diachylon, to active pommelling with the fists. But we rather think common sense will be found quite adequate to its due regulation, without the aid of such companions of his "échafaudage de lignes."* 7. O'Beirne's

* A most important improvement in the mode of applying pressure to diseased parts has been recently made by Dr. Arnott. In this plan, pressure is applied on the *hydro-*

mode of treating diseased joints by calomel and opium to salivation is alluded to, and censured as too indiscriminately employed. We agree fully on this point; and have no hesitation in stating our firm belief that, while Dr. O'Beirne has done much good service to his profession and humanity by introducing this remedy, as one of the most valuable means whereby to arrest *acute* affections of joints, he has almost, if not wholly, neutralized this benefit by pushing it too far; extending its use to chronic affections even of an obviously scrofulous nature, in which amendment, if it occur at all thus, can only be purchased—blindly purchased—at the cost of future misery and destruction. It is the way with almost all new remedies. The unwise zeal of their happy and fond and doating parents carries them too fast and too far; disappointment, doubt, distrust, vacillation result; and some time is required ere the panacea, tumbled from the high perch where it ought never to have been, can settle down into its just and proper place. 8. As a local discutient, M. Lisfranc prefers the iodide of lead to the hydriodate of potash, the latter being apt to create an unnecessary disturbance on the surface; and, in regard to the use of all such applications, he wisely recommends caution, lest they overdo their work and bring on inflammation into all the diseased structure. As an internal remedy, the hydriodate of potass receives its due meed of commendation. 9. Another good general rule is enforced connected with the counter-irritation: never to place the cautery, moxa, seton, &c. too near the diseased part, not on the swelling, but in its immediate vicinity, and never to employ such remedies during the period of actual inflammation, otherwise the effect must be the reverse of beneficial. 10. The muriate of barytes, as an antagonist of everything scrofulous and of anything seeming to appertain thereunto, has long fallen into disrepute in this country, failure and disappointment having all but uniformly followed upon its use. M. Pirondi, of Marseilles, thinks otherwise; and in M. Lisfranc it has found a most powerful advocate, and one who by no niggardly praise would wish to push it foremost in the list of means wherewith to attack the formidable *omnium gatherum* which is designated by him "white swelling." The dose is a gradual ascension from gr. vi. to ʒij, with a non-stimulant vegetable system of diet, and with a guarantee as to the non-occurrence of debility therefrom: on the contrary. It is admitted, however, that symptoms of poisoning sometimes threaten; and to arrest these, the white of an egg is said to act as by enchantment. In support of his opinion, a considerable number of cases are brought forward; but we need scarcely say that the evidence therein contained, to establish the efficacy of this medicine, is by no means satisfactory. *Besides* the barytes, we read of pressure, counter-irritation, hydriodate of potass, mercury outside and in, leeches, rest, &c. in the treatment; and surely these, aggregately, if not singly, are at least as much entitled to the credit of the cure. On this point our author again finds himself at loggerheads with M. Velpeau; and, notwithstanding an eloquent statement on the other side, we are inclined to take part with the latter, even at the risk of being told that we have "not a single sound idea of therapeutics within our cranium." A catalogue raisonné of the good effects of the muriate is given at p. 627 et seq., but we care not to transcribe them. 11. A recovered joint gets

*static principle.* Most extraordinary results have been already obtained from it in the case of tumours of the mamma.—Ed.

CHARITY HOSPITAL, MEDICAL LIBRARY.

back its functions but gradually; and it is folly, or worse, to expect sudden amendment in this particular. A useful practical rule is given on this subject. Pain induced by motion and exercise, and disappearing within half an hour after resumption of repose, does not necessarily indicate a necessity for continuance of undisturbed rest; but if the patient continue to suffer, rest and sedatives are still essential. 12. M. Lisfranc believes that there is no pathognomonic signs of true anchylosis having occurred, and therefore advises gradual and gentle attempts to restore normal motion and position to all stiff joints otherwise recovered from disease. The barbarous employment of brute force towards this end, as practised by M. Louvrier, is justly condemned; but on this subject we will not enter, being afraid lest our usual serenity of temper desert us on the occasion.

*Some remarks on exostosis—not venereal.* In the term exostosis M. Lisfranc seems to include all tumours or enlargements of bone; not limiting it, as most surgeons here do, to mere local hypertrophy or simple enlargement of that texture. 1. That enlargements of bone can and do occur without the assistance of either "vice or virus," is a statement the truth of which we most willingly attest. And we believe that if this were more generally felt and acted on, we should, by so escaping at least some cases of blind mercurialism, have fewer of the worst class of osseous enlargements, and fewer samples of a hopelessly riven and shattered frame. It is consistent with our own knowledge that there are still men in the profession who hold that all bony swellings are nodes, that all nodes are venereal, and that all venereal nodes are to be treated by mercury, and nothing but mercury. "Heaven mend their wits!" 2. A tumour of the soft parts resting on bone involves this texture in disease; and this disease may be either simple or similar in character and disposition to that of the originating growth. In the latter case the bone, or at all events, the implicated portion of it, is to be taken away along with the tumour; in the former it is left behind, at least in the first instance; a chance being afforded it of recovery to a sound condition, and of which chance it will usually be found to take a commendable advantage. The favorable or unfavorable condition can only be ascertained by most careful examination, both of the exposed bone and of the primitive tumour. In all cases of doubt we would haste to err on the safe side, by at once removing the implicated bone, being well aware that secondary formations of tumour, whether in the soft or hard tissues, are almost invariably worse than their predecessors. 3. Ordinary bruises of bone, by inducing a perverted nutrition in that texture, may lead to the most serious morbid products. M. Lisfranc complains that this undeniable truth is not sufficiently borne in mind in the usual treatment, immediate and secondary, of such accidents. Often the apparently simple bruise is neglected and forgotten, until the formidable caries, necrosis, or tumour has made an obvious demonstration.

And now, having overtaken all the points which appeared to us deserving of notice, whether for censure or for praise, we close the volume, with undiminished feelings of respect for its author, but with a caution to all intending readers to beware how they lend implicit faith to all the doctrines, and blindly follow all the precepts, even of this Coryphæus of his art.

